# First 1000 Days of Life: Consequences of Antibiotics on Gut Microbiota

**DOI:** 10.3389/fmicb.2021.681427

**Published:** 2021-05-19

**Authors:** Julio Aires

**Affiliations:** ^1^Université de Paris, INSERM, UMR-S1139 (3PHM), Paris, France; ^2^FHU PREMA, Hôpital Cochin, Paris, France

**Keywords:** antibiotics, gut microbiota, DOHaD, health consequences, resistome

## Abstract

The developmental origin of health and disease highlights the importance of the period of the first 1000 days (from conception to 2 years) of life. In particular, the process of gut microbiota establishment occurs within this time window. Therefore, determinants interfering with neonatal gut establishment may disrupt its physiological functions and potentially lead to negative health outcomes. Antibiotics are among perinatal determinants that can directly or indirectly affect the pattern of gut bacterial colonization, with a long-lasting impact on intestinal ecosystem functions. In this review, we will examine the impact of antibiotics on the intestinal microbiota during the perinatal period and first years of life, a key interval for development of an individual’s health capital. Further, we will discuss the role of antibiotics during short- and long-term dysbiosis and their associated health consequences.

## Introduction

The human digestive microbial ecosystem represents a considerable biomass with multiple roles, including metabolic, barrier, and immune functions. Establishment of the intestinal microbiota is crucial for these functions. The developmental origin of health and disease, or DOHaD, emphasizes the importance of the first 1000 days, from conception to the 2 years of life, for future health. This concept includes microbiota establishment, particularly the intestinal microbiota, whose impact on the possible occurrence of subsequent pathologies is becoming increasingly evident (Butel et al., 2018). Further, because multiple processes are regulated by the intestinal microbiota, any microbial imbalance can impact its physiological functions and the health of the host.

**FIGURE 1:**
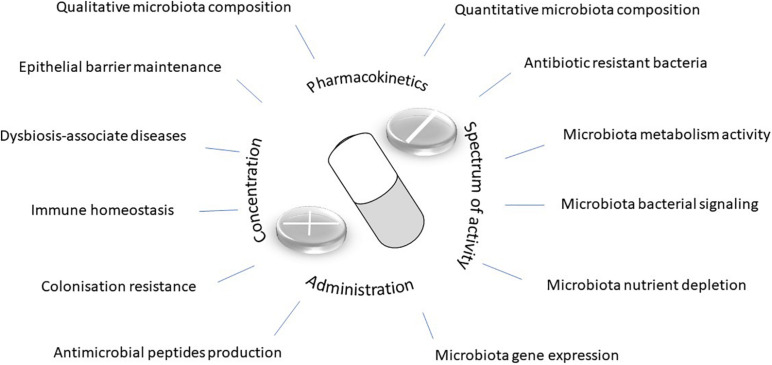
Antibiotic impact.

Natural or synthetic organic substances and antibiotics are defined by their ability to limit or prevent proliferation of pathogenic bacteria. Since the first reported use of sulfanilamide and penicillin in humans, evidence of the efficacy of antibiotic treatment for infectious diseases has been widely established, including significant reductions in the mortality rate (Rustam, 2010). In recent years, multiple studies have shown that over-consumption, prolonged use, incorrect dosage, or the pharmacological properties of antibiotics can have unforeseen and undesirable consequences on the intestinal microbiota (Butel et al., 2018). The success of antibiotic therapy, whether curative or prophylactic, is based on microbiological, pharmacological (pharmacokinetics and pharmacodynamics), and clinical characteristics of the antibiotics used in treatment. Antibiotic effects on the intestinal microbiota are dependent on the spectrum, doses, duration of treatment, route of administration, and pharmacokinetic and pharmacodynamic properties of the considered molecule (Figure 1). In this review, we will focus on the impact of antibiotics on the intestinal microbiota during the perinatal period and first years of life, an important phase during the development of an individual’s health capital. Additionally, we will discuss the role of antibiotics in short- and long-term dysbiosis and their health consequences.

## Antibiotics

The perinatal period represents the first window of potential antibiotic exposure in humans. This period is characterized by a high incidence of neonatal infections, which are the leading cause of death (Thea and Qazi, 2008), and corresponds to an increased risk of exposure to nosocomial infection in hospitalized newborns. During this period, the absence of markers or symptoms suggestive of infection makes it difficult to establish a diagnosis, resulting in an early and probabilistic prescription. In industrialized countries, more than 50% of children have been prescribed antibiotics during their first year of life (Nogacka et al., 2018). Despite the existence of guidelines concerning the diagnosis and curative treatment of bacterial neonatal infections (Fuchs et al., 2018), significant heterogeneity in the management of suspected infections has been observed. Specifically, variability in the practice of antibiotic prescriptions in neonatology has been demonstrated both nationally and internationally. For example, in the United States, variations (up to 40-fold) in dosing regimens were observed across 127 neonatal intensive care units with equivalent levels of infection (Schulman et al., 2015). In France, following analysis of antibiotic prescription protocols used in 44 neonatal intensive care units, it was shown that 41 antibiotic molecules were identified, with an average of nine different dosage regimens per molecule. Additionally, significant variations in daily doses were found, depending on the protocols (Leroux et al., 2015). In the United Kingdom, Kadambari et al. (2011) identified 10 different dosing regimens for gentamicin prescriptions in 43 neonatal units. In addition to the variability in the application of dosing recommendations, the use of non-authorized drugs has also been reported (Magalhães et al., 2015).

## Intestinal Microbiota: Impact of Antibiotics

In adults, antibiotics are known to cause temporary changes to microbiota, without any recognized consequences in most cases, except for the emergence of bacterial resistance. A relatively rapid return to the previous microbiota profile is possible owing to its functional redundancy or resilience (Dethlefsen and Relman, 2011). However, perinatal disturbances during establishment of the gut microbiota, which is less diverse than adults, can lead to modifications that prevent resilience and disrupt the essential functions of the gut (Figure 1).

Early antibiotic therapy decreases the biodiversity of neonatal microbiota, including decreases in beneficial genera, such as *Bifidobacterium* and *Lactobacillus* (Tanaka et al., 2009; Fouhy et al., 2012), or decreases in the abundance of *Clostridiales* (including *Lachnospiraceae*) and *Ruminococcus* (Fjalstad et al., 2018). Few studies have analyzed the impact on microbiota relative to the duration of antibiotic therapy. In neonates treated for less than 72 h for suspected unconfirmed infection, a decrease in bacterial diversity was associated with prolonged antibiotic therapy (Fjalstad et al., 2018). Gut bacterial groups can be modified during the first 2–3 years of life, such as *Lachnospiraceae*, which are butyrate-producing bacteria, a metabolite involved in the maturation of the intestinal mucosa (Bokulich et al., 2016). In a study by Yassour et al. (2016), antibiotic therapy during the first year of life was found to be associated with a decrease in microbial diversity at the age of 3 years.

Very preterm or very low birth weight infants are at a higher risk of dysbiosis. They receive more frequent broad-spectrum probabilistic treatment at birth. They also have delayed bacterial colonization kinetics in the gut, resulting in a reduced number of bacterial species in the intestinal microbiota (Arboleya et al., 2013; La Rosa et al., 2014). Early empiric antibiotic use in preterm infants is associated with a decrease in bacterial diversity (Greenwood et al., 2014). Bacterial equilibrium is also altered by an increase in enterobacteria and a decrease in potentially beneficial bacterial groups, such as *Bifidobacterium*, *Bacilli*, and *Lactobacillales* (Greenwood et al., 2014; Zwittink et al., 2018). Premature infants either untreated (*n* = 5), treated for less than 3 days (*n* = 5), or treated for 5 days or more (*n* = 5) showed a decrease in bifidobacteria and enterobacteria, at the expense of enterococci (Zwittink et al., 2018). However, no impact on diversity was observed. Interestingly, short-term treatment allowed a return to a similar profile in untreated newborns within 3 weeks, whereas changes in the profile were still visible after 6 weeks in the long-term treatment groups.

## Maternal Antibiotics

Maternal antibiotic therapy can affect the development of the neonatal microbiota. The consensual practice of intrapartum antibiotic prophylaxis treatment against neonatal group B streptococcal infection has proven to be effective in reducing its incidence infections. In industrialized countries, approximately 30% of women receive antibiotic prophylaxis, making it the main cause of perinatal antibiotic exposure (Nogacka et al., 2018). Several studies investigating the impact of this treatment on microbiota establishment have shown a decrease in colonization of some genera (Arboleya et al., 2015; Aloisio et al., 2016). Children born to mothers who received intrapartum antibiotic therapy showed less colonization by bacteria belonging to the phylum *Actinobacteria* (including *Bifidobacterium*) or genus *Lactobacillus*, and were more frequently colonized by the phyla *Firmicutes* and *Proteobacteria* (including *Enterobacteriaceae*). Over time, changes are still visible during the first months of life (Azad et al., 2016; Nogacka et al., 2017).

Vaginal and fecal microbiota play a role in the microbiota establishment in newborns via vertical transmission (Dominguez-Bello et al., 2010; Sakwinska et al., 2017). The vaginal microbiota can evolve during pregnancy (Kervinen et al., 2019). Antibiotic therapies during pregnancy, especially at the end of pregnancy to treat premature rupture of membranes, can alter the microbiota and thus the bacterial establishment in the unborn child (Walther-António et al., 2014).

## Antibiotics: Pathophysiological Consequences or Epidemiological Association

### Short-Term Consequences

Establishment and maturation of the microbiota in newborns are important physiological steps. Early dysbiosis can result in short- and long-term health consequences. Regarding short-term consequences, decreases in the phylogenetic diversity of intestinal microbiota following antibiotic treatment have been correlated with increased frequency of sepsis in premature infants (Madan et al., 2012; Carl et al., 2014). Similarly, broad-spectrum antibiotic therapy has been associated with an increased risk of developing necrotizing enterocolitis in premature infants (Kuppala et al., 2011; Cotten, 2016).

### Long-Term Consequences

For long-term consequences, early exposure to antibiotics that leads to dysbiosis is a risk factor for pathologies associated with poor maturation of the immune system and impaired metabolic functions of the microbiota. Antibiotic exposure in young children may also lead to the development of pathologies associated with immune disorders, such as asthma, allergic diseases, or eczema. Several studies, mostly on cohort follow-ups of children, have reported an association between pre- and postnatal exposure to antibiotics and an increased risk of developing asthma in early childhood (Murk et al., 2011; Heintze and Petersen, 2013; Metsälä et al., 2015; Pitter et al., 2016). The risk of developing asthma after antibiotic therapy, whether prenatal, perinatal, or during the first year of life, was frequently observed [odds ratio (OR) = 1.2–2]. In the follow-up of a large Swedish cohort, the inclusion of siblings as a control population showed a decreased, yet significant (OR = 2.36), probability of this association with respiratory infection in early childhood, but the association disappeared when considering antibiotic therapy during pregnancy. This suggests an important role of the familial environment in allergy development. Another study described a weak relationship (OR = 2.3) between antibiotic therapy during the first year and asthma, but no association was found at the age of 5 years with subsequent occurrences of recurrent asthma, eczema, or immunological criteria of atopy (Kusel et al., 2008). Additionally, a meta-analysis of 22 studies found a relationship between antibiotic exposure during the first 2 years of life and the subsequent risk of eczema (OR = 1.26) or hay fever (OR = 1.23), and for three of these studies, an increased risk of developing food allergy (OR = 1.42) (Ahmadizar et al., 2018). However, no association was found with objective measures of atopy, such as prick tests and specific IgE levels.

Links between early and/or repeated exposure to antibiotics and the risk of developing Crohn’s disease or inflammatory bowel disease (IBD) have been established (Shaw et al., 2010; Hviid et al., 2011). In a meta-analysis of 11 studies looking for an association between the accumulation of antibiotic exposure and subsequent risk of IBD, a significant association was found for the occurrence of Crohn’s disease but not for ulcerative colitis (Ungaro et al., 2014), which was higher in pediatric patients with Crohn’s disease. A Danish cohort study also demonstrated an increased risk of pediatric Crohn’s disease after multiple antibiotic treatments (relative risk = 7.32, in children who received 7 or more courses of antibiotics); however, the causal relationship needs to be confirmed as antibiotic therapy may serve as a marker for the management of digestive symptoms in undiagnosed Crohn’s disease (Hviid et al., 2011). Further, a Swedish cohort study showed an association between antibiotic therapy during pregnancy in the third trimester and an increased risk of infantile Crohn’s disease (OR = 2.48). Yet, this association was not found for antibiotic treatment prescribed in early childhood or ulcerative colitis (Örtqvist et al., 2014). In the field of digestive disorders, a significant association between planned cesarean section birth and the subsequent occurrence of celiac disease should be noted (OR = 1.15) (Mårild et al., 2013).

Obesity has been associated with low diversity and an abnormal profile of the adult microbiota (Le Chatelier et al., 2013). Several studies have shown an association between exposure to antibiotics during the first year of life and obesity in children (Trasande et al., 2013; Azad et al., 2014; Bailey et al., 2014; Murphy et al., 2014). This association appears to be stronger in boys than in girls (Azad et al., 2014; Murphy et al., 2014). A study on a US cohort of more than 360,000 children found a relationship between being overweight at age 5 and exposure to antibiotics during the first 2 years of life (Block et al., 2018). Moreover, two reviews and meta-analyses have highlighted the association between early and/or repeated exposure to antibiotics and an increased risk of being overweight or obese, even though this increase is relatively small (Miller et al., 2018; Rasmussen et al., 2018). Although the causal relationship between alterations in the microbiota composition and antibiotics has not been formally demonstrated, the microbiota is known to increase the capacity of energy storage from food. In addition, certain bacterial genera or groups have been implicated in low-grade inflammation in obesity (Gasparrini et al., 2016).

## Resistome

A set of resistance genes is present in the intestinal microbiota, representing the endogenous or resident resistome. It is composed of resistance genes from the host’s resident microbiota and by exogenous or variable genes from bacteria in transit (Ruppé et al., 2016). Studies of the resistome have been carried out using culture, PCR, or metagenomics approaches. The microbiota of newborns hosts a large variety of antibiotic resistance genes (Francino, 2015; Moore et al., 2015). Gosalbes et al. (2016) have shown that many antibiotic resistance genes can be identified in fecal samples of one-week-old newborns, and even in meconium samples. Antibiotic resistance genes are detected not only in adults and children who have undergone antibiotic treatment, but also in newborns who are naïve to antibiotic treatment, possibly due to vertical maternal transmission of these genes (Vaishampayan et al., 2010; Moore et al., 2015). Some resistance genes may be common between maternal and neonatal fecal samples. In some cases, common resistance genes are present in meconium, colostrum, or maternal milk samples (Francino, 2015). However, resistance genes that are absent in mothers have been detected in neonates, reflecting acquisition from other sources. Indeed, reports have highlighted the presence of antibiotic-resistant bacteria and genes into the environment, including hospitals, and therefore transmissible (Kim and Cha, 2021). Besides, the abundance of resistance genes was also correlated with antibiotic use practices. Yassour et al. (2016) showed an increase in resistance genes following antibiotic administration, which decreased after cessation of treatment, while the mobile genetic elements of resistance remained high. For intrapartum group B streptococcal antibiotic prophylaxis, an increase in resistance of 30–50% to erythromycin and 25–35% to clindamycin has been observed (Verani et al., 2010). Moreover, intrapartum antibiotic therapy has been associated with increased ampicillin-resistant *Escherichia coli* in premature infants (Stoll et al., 2002; Bizzarro and Gallagher, 2007; Bizzarro et al., 2008; Kuhn et al., 2010). The combination of cefotaxime-amoxicillin and penicillin-tobramycin increases the relative risk of colonization with a resistant strain by a factor of 18 (de Man et al., 2000). Further, an extended duration of antibiotic therapy has been shown as a risk factor for the development of late neonatal infection (Didier et al., 2012).

## Conclusion

Data on the impact of antibiotic therapy on the newborn intestinal microbiota clearly show an alteration in bacterial load and diversity. The effect of antibiotics appears to be long-lasting, with a rapid return to the original composition of the microbiota, depending on the antibiotic molecule. The consequences of modifying the microbiota balance include an increased risk of developing some pathologies later in life and bacterial resistance. This underlines the importance of the rational use of antibiotics, particularly choosing among the recommended antibiotics, giving preference to the narrowest spectrum molecules to minimize side effects. Antibiotic stewardship should make sense. While it is clear that antibiotics influence the establishment of the intestinal microbiota, the parameters associated with antibiotic use, such as specificity, dose, treatment time, and mode of administration, have not been studied, making it difficult to accurately determine the precise impact of antibiotics.

## Author Contributions

The author confirms being the sole contributor of this work and has approved it for publication.

## Conflict of Interest

The author declares that the research was conducted in the absence of any commercial or financial relationships that could be construed as a potential conflict of interest.

## References

[B1] Ahmadizar F. Vijverberg S. J. H. Arets H. G. M. de Boer A. Lang J. E. Garssen J. (2018). Early-life antibiotic exposure increases the risk of developing allergic symptoms later in life: a meta-analysis. *Allergy* 73 971–986. 10.1111/all.13332 29105784

[B2] Aloisio I. Quagliariello A. De Fanti S. Luiselli D. De Filippo C. Albanese D. (2016). Evaluation of the effects of intrapartum antibiotic prophylaxis on newborn intestinal microbiota using a sequencing approach targeted to multi hypervariable 16S rDNA regions. *Appl. Microbiol. Biotechnol.* 100 5537–5546. 10.1007/s00253-016-7410-2 26971496

[B3] Arboleya S. Salazar N. Solís G. Fernández N. Hernández-Barranco A. M. Cuesta I. (2013). Assessment of intestinal microbiota modulation ability of *Bifidobacterium* strains in in vitro fecal batch cultures from preterm neonates. *Anaerobe* 19 9–16. 10.1016/j.anaerobe.2012.11.001 23154045

[B4] Arboleya S. Sánchez B. Milani C. Duranti S. Solís G. Fernández N. (2015). Intestinal microbiota development in preterm neonates and effect of perinatal antibiotics. *J. Pediatr.* 166 538–544. 10.1016/j.jpeds.2014.09.041 25444008

[B5] Azad M. B. Bridgman S. L. Becker A. B. Kozyrskyj A. L. (2014). Infant antibiotic exposure and the development of childhood overweight and central adiposity. *Int. J. Obes.* 38 1290–1298. 10.1038/ijo.2014.119 25012772

[B6] Azad M. B. Konya T. Persaud R. R. Guttman D. S. Chari R. S. Field C. J. (2016). Impact of maternal intrapartum antibiotics, method of birth and breastfeeding on gut microbiota during the first year of life: a prospective cohort study. *BJOG* 123 983–993. 10.1111/1471-0528.13601 26412384

[B7] Bailey L. C. Forrest C. B. Zhang P. Richards T. M. Livshits A. DeRusso P. A. (2014). Association of antibiotics in infancy with early childhood obesity. *JAMA Pediatr.* 168 1063–1069. 10.1001/jamapediatrics.2014.1539 25265089

[B8] Bizzarro M. J. Dembry L. M. Baltimore R. S. Gallagher P. G. (2008). Changing patterns in neonatal *Escherichia coli* sepsis and ampicillin resistance in the era of intrapartum antibiotic prophylaxis. *Pediatrics* 121 689–696. 10.1542/peds.2007-2171 18381532

[B9] Bizzarro M. J. Gallagher P. G. (2007). Antibiotic-resistant organisms in the neonatal intensive care unit. *Semin. Perinatol.* 31 26–32. 10.1053/j.semperi.2007.01.004 17317424

[B10] Block J. P. Bailey L. C. Gillman M. W. Lunsford D. Daley M. F. Eneli I. (2018). Early antibiotic exposure and weight outcomes in young children. *Pediatrics* 142:e20180290. 10.1542/peds.2018-0290 30381474 PMC6317759

[B11] Bokulich N. A. Chung J. Battaglia T. Henderson N. Jay M. Li H. (2016). Antibiotics, birth mode, and diet shape microbiome maturation during early life. *Sci. Transl. Med.* 8:343ra82. 10.1126/scitranslmed.aad7121 27306664 PMC5308924

[B12] Butel M. J. Waligora-Dupriet A. J. Wydau-Dematteis S. (2018). The developing gut microbiota and its consequences for health. *J. Dev. Orig. Health Dis.* 9 590–597. 10.1017/S2040174418000119 29562949

[B13] Carl M. A. Ndao I. M. Springman A. C. Manning S. D. Johnson J. R. Johnston B. D. (2014). Sepsis from the gut: the enteric habitat of bacteria that cause late-onset neonatal bloodstream infections. *Clin. Infect. Dis.* 58 1211–1218. 10.1093/cid/ciu084 24647013 PMC3982840

[B14] Cotten C. M. (2016). Adverse consequences of neonatal antibiotic exposure. *Curr. Opin. Pediatr.* 28 141–149. 10.1097/MOP.0000000000000338 26886785 PMC4845665

[B15] de Man P. Verhoeven B. A. Verbrugh H. A. Vos M. C. van den Anker J. N. (2000). An antibiotic policy to prevent emergence of resistant bacilli. *Lancet* 355 973–978. 10.1016/s0140-6736(00)90015-1 10768436

[B16] Dethlefsen L. Relman D. A. (2011). Incomplete recovery and individualized responses of the human distal gut microbiota to repeated antibiotic perturbation. *Proc. Natl. Acad. Sci. U.S.A.* 108 4554–4561. 10.1073/pnas.1000087107 20847294 PMC3063582

[B17] Didier C. Streicher M. P. Chognot D. Campagni R. Schnebelen A. Messer J. (2012). Late-onset neonatal infections: incidences and pathogens in the era of antenatal antibiotics. *Eur. J. Pediatr.* 171 681–687. 10.1007/s00431-011-1639-7 22134805

[B18] Dominguez-Bello M. G. Costello E. K. Contreras M. Magris M. Hidalgo G. Fierer N. (2010). Delivery mode shapes the acquisition and structure of the initial microbiota across multiple body habitats in newborns. *Proc. Natl. Acad. Sci. U.S.A.* 107 11971–11975. 10.1073/pnas.1002601107 20566857 PMC2900693

[B19] Fjalstad J. W. Esaiassen E. Juvet L. K. van den Anker J. N. Klingenberg C. (2018). Antibiotic therapy in neonates and impact on gut microbiota and antibiotic resistance development: a systematic review. *J. Antimicrob. Chemother.* 73 569–580. 10.1093/jac/dkx426 29182785

[B20] Fouhy F. Guinane C. M. Hussey S. Wall R. Ryan C. A. Dempsey E. M. (2012). High-throughput sequencing reveals the incomplete, short-term recovery of infant gut microbiota following parenteral antibiotic treatment with ampicillin and gentamicin. *Antimicrob. Agents Chemother.* 56 5811–5820. 10.1128/AAC.00789-12 22948872 PMC3486619

[B21] Francino M. P. (2015). Antibiotics and the human gut microbiome: dysbioses and accumulation of resistances. *Front. Microbiol.* 6:1543. 10.3389/fmicb.2015.01543 26793178 PMC4709861

[B22] Fuchs A. Bielicki J. Mathur S. Sharland M. van den Anker J. N. (2018). Reviewing the WHO guidelines for antibiotic use for sepsis in neonates and children. *Paediatr. Int. Child Health* 38 S3–S15. 10.1080/20469047.2017.1408738 29790842 PMC6176768

[B23] Gasparrini A. J. Crofts T. S. Gibson M. K. Tarr P. I. Warner B. B. Dantas G. (2016). Antibiotic perturbation of the preterm infant gut microbiome and resistome. *Gut Microbes* 7 443–449. 10.1080/19490976.2016.1218584 27472377 PMC5154371

[B24] Gosalbes M. J. Vallès Y. Jiménez-Hernández N. Balle C. Riva P. Miravet-Verde S. (2016). High frequencies of antibiotic resistance genes in infants’ meconium and early fecal samples. *J. Dev. Orig. Health Dis.* 7 35–44. 10.1017/S2040174415001506 26353938

[B25] Greenwood C. Morrow A. L. Lagomarcino A. J. Altaye M. Taft D. H. Yu Z. (2014). Early empiric antibiotic use in preterm infants is associated with lower bacterial diversity and higher relative abundance of *Enterobacter*. *J. Pediatr.* 165 23–29. 10.1016/j.jpeds.2014.01.010 24529620 PMC4074569

[B26] Heintze K. Petersen K. U. (2013). The case of drug causation of childhood asthma: antibiotics and paracetamol. *Eur. J. Clin. Pharmacol.* 69 1197–1209. 10.1007/s00228-012-1463-7 23292157 PMC3651816

[B27] Hviid A. Svanström H. Frisch M. (2011). Antibiotic use and inflammatory bowel diseases in childhood. *Gut* 60 49–54. 10.1136/gut.2010.219683 20966024

[B28] Kadambari S. Heath P. T. Sharland M. Lewis S. Nichols A. Turner M. A. (2011). Variation in gentamicin and vancomycin dosage and monitoring in UK neonatal units. *J. Antimicrob. Chemother.* 66 2647–2650. 10.1093/jac/dkr351 21862473

[B29] Kervinen K. Kalliala I. Glazer-Livson S. Virtanen S. Nieminen P. Salonen A. (2019). Vaginal microbiota in pregnancy: role in induction of labor and seeding the neonate’s microbiota? *J Biosci*. 44:116. 31719225

[B30] Kim D. W. Cha C. J. (2021). Antibiotic resistome from the One-Health perspective: understanding and controlling antimicrobial resistance transmission. *Exp. Mol. Med*. 53 301–309. 10.1038/s12276-021-00569-z 33642573 PMC8080597

[B31] Kuhn P. Dheu C. Bolender C. Chognot D. Keller L. Demil H. (2010). Incidence and distribution of pathogens in early-onset neonatal sepsis in the era of antenatal antibiotics. *Paediatr. Perinat. Epidemiol.* 24 479–487. 10.1111/j.1365-3016.2010.01132.x 20670228

[B32] Kuppala V. S. Meinzen-Derr J. Morrow A. L. Schibler K. R. (2011). Prolonged initial empirical antibiotic treatment is associated with adverse outcomes in premature infants. *J. Pediatr.* 159 720–725. 10.1016/j.jpeds.2011.05.033 21784435 PMC3193552

[B33] Kusel M. M. de Klerk N. Holt P. G. Sly P. D. (2008). Antibiotic use in the first year of life and risk of atopic disease in early childhood. *Clin. Exp. Allergy* 38 1921–1928. 10.1111/j.1365-2222.2008.03138.x 19037966

[B34] La Rosa P. S. Warner B. B. Zhou Y. Weinstock G. M. Sodergren E. Hall-Moore C. M. (2014). Patterned progression of bacterial populations in the premature infant gut. *Proc. Natl. Acad. Sci. U.S.A.* 111 12522–12527. 10.1073/pnas.1409497111 25114261 PMC4151715

[B35] Le Chatelier E. Nielsen T. Qin J. Prifti E. Hildebrand F. Falony G. (2013). Richness of human gut microbiome correlates with metabolic markers. *Nature* 500 541–546. 10.1038/nature12506 23985870

[B36] Leroux S. Zhao W. Bétrémieux P. Pladys P. Saliba E. Jacqz-Aigrain E. (2015). Therapeutic guidelines for prescribing antibiotics in neonates should be evidence-based: a French national survey. *Arch. Dis. Child.* 100 394–398. 10.1136/archdischild-2014-306873 25628457

[B37] Madan J. C. Salari R. C. Saxena D. Davidson L. O’Toole G. A. Moore J. H. (2012). Gut microbial colonisation in premature neonates predicts neonatal sepsis. *Arch. Dis. Child. Fetal Neonat. Ed.* 97 F456–F462. 10.1136/fetalneonatal-2011-301373 22562869 PMC3724360

[B38] Magalhães J. Rodrigues A. T. Roque F. Figueiras A. Falcão A. Herdeiro M. T. (2015). Use of off-label and unlicenced drugs in hospitalised paediatric patients: a systematic review. *Eur. J. Clin. Pharmacol.* 71 1–13. 10.1007/s00228-014-1768-9 25318905

[B39] Mårild K. Ye W. Lebwohl B. Green P. H. Blaser M. J. Card T. (2013). Antibiotic exposure and the development of coeliac disease: a nationwide case-control study. *BMC Gastroenterol.* 13:109. 10.1186/1471-230X-13-109 23834758 PMC3720284

[B40] Metsälä J. Lundqvist A. Virta L. J. Kaila M. Gissler M. Virtanen S. M. (2015). Prenatal and post-natal exposure to antibiotics and risk of asthma in childhood. *Clin. Exp. Allergy* 45 137–145. 10.1111/cea.12356 24943808

[B41] Miller S. A. Wu R. K. S. Oremus M. (2018). The association between antibiotic use in infancy and childhood overweight or obesity: a systematic review and meta-analysis. *Obes. Rev.* 19 1463–1475. 10.1111/obr.12717 30035851

[B42] Moore A. M. Ahmadi S. Patel S. Gibson M. K. Wang B. Ndao M. I. (2015). Gut resistome development in healthy twin pairs in the first year of life. *Microbiome* 3:27. 10.1186/s40168-015-0090-9 26113976 PMC4480905

[B43] Murk W. Risnes K. R. Bracken M. B. (2011). Prenatal or early-life exposure to antibiotics and risk of childhood asthma: a systematic review. *Pediatrics* 127 1125–1138. 10.1542/peds.2010-2092 21606151

[B44] Murphy R. Stewart A. W. Braithwaite I. Beasley R. Hancox R. J. Mitchell E. A. (2014). Antibiotic treatment during infancy and increased body mass index in boys: an international cross-sectional study. *Int. J. Obes.* 38 1115–1119. 10.1038/ijo.2013.218 24257411

[B45] Nogacka A. Salazar N. Suárez M. Milani C. Arboleya S. Solís G. (2017). Impact of intrapartum antimicrobial prophylaxis upon the intestinal microbiota and the prevalence of antibiotic resistance genes in vaginally delivered full-term neonates. *Microbiome* 5:93. 10.1186/s40168-017-0313-3 28789705 PMC5549288

[B46] Nogacka A. M. Salazar N. Arboleya S. Suárez M. Fernández N. Solís G. (2018). Early microbiota, antibiotics and health. *Cell. Mol. Life Sci.* 75 83–91. 10.1007/s00018-017-2670-2 28988290 PMC11105232

[B47] Örtqvist A. K. Lundholm C. Kieler H. Ludvigsson J. F. Fall T. Ye W. (2014). Antibiotics in fetal and early life and subsequent childhood asthma: nationwide population based study with sibling analysis. *BMJ* 349:g6979. 10.1136/bmj.g6979 25432937 PMC4247260

[B48] Pitter G. Ludvigsson J. F. Romor P. Zanier L. Zanotti R. Simonato L. (2016). Antibiotic exposure in the first year of life and later treated asthma, a population based birth cohort study of 143,000 children. *Eur. J. Epidemiol.* 31 85–94. 10.1007/s10654-015-0038-1 25957084

[B49] Rasmussen S. H. Shrestha S. Bjerregaard L. G. Ängquist L. H. Baker J. L. Jess T. (2018). Antibiotic exposure in early life and childhood overweight and obesity: a systematic review and meta-analysis. *Diabetes Obes. Metab.* 20 1508–1514. 10.1111/dom.13230 29359849

[B50] Ruppé E. Le Chatelier E. Pons N. Andremont A. Ehrlich S. D. (2016). Dissemination of the mcr-1 colistin resistance gene. *Lancet Infect. Dis.* 16 290–291. 10.1016/S1473-3099(16)00066-9 26973305

[B51] Rustam I. A. (2010). A brief history of the antibiotic era: lessons learned and challenges for the future. *Front. Microbiol.* 1:134. 10.3389/fmicb.2010.00134 21687759 PMC3109405

[B52] Sakwinska O. Foata F. Berger B. Brüssow H. Combremont S. Mercenier A. (2017). Does the maternal vaginal microbiota play a role in seeding the microbiota of neonatal gut and nose? *Benef. Microbes* 8 763–778. 10.3920/BM2017.0064 29022384

[B53] Schulman J. Dimand R. J. Lee H. C. Duenas G. V. Bennett M. V. Gould J. B. (2015). Neonatal Intensive Care Unit antibiotic use. *Pediatrics* 135 826–833. 10.1542/peds.2014-3409 25896845

[B54] Shaw S. Y. Blanchard J. F. Bernstein C. N. (2010). Association between the use of antibiotics in the first year of life and pediatric inflammatory bowel disease. *Am. J. Gastroenterol.* 105 2687–2692. 10.1038/ajg.2010.398 20940708

[B55] Stoll B. J. Hansen N. Fanaroff A. A. Wright L. L. Carlo W. A. Ehrenkranz R. A. (2002). Changes in pathogens causing early-onset sepsis in very-low-birth-weight infants. *N. Engl. J. Med.* 347 240–247. 10.1056/NEJMoa012657 12140299

[B56] Tanaka S. Kobayashi T. Songjinda P. Tateyama A. Tsubouchi M. Kiyohara C. (2009). Influence of antibiotic exposure in the early postnatal period on the development of intestinal microbiota. *FEMS Immunol. Med. Microbiol.* 56 80–87. 10.1111/j.1574-695X.2009.00553.x 19385995

[B57] Thea D. Qazi S. (2008). Neonatal mortality - 4 million reasons for progress. *Lancet* 371 1893–1895. 10.1016/S0140-6736(08)60810-7 18539207

[B58] Trasande L. Blustein J. Liu M. Corwin E. Cox L. M. Blaser M. J. (2013). Infant antibiotic exposures and early-life body mass. *Int. J. Obes.* 37 16–23. 10.1038/ijo.2012.132 22907693 PMC3798029

[B59] Ungaro R. Bernstein C. N. Gearry R. Hviid A. Kolho K. L. Kronman M. P. (2014). Antibiotics associated with increased risk of new-onset Crohn’s disease but not ulcerative colitis: a meta-analysis. *Am. J. Gastroenterol.* 109 1728–1738. 10.1038/ajg.2014.246 25223575

[B60] Vaishampayan P. A. Kuehl J. V. Froula J. L. Morgan J. L. Ochman H. Francino M. P. (2010). Comparative metagenomics and population dynamics of the gut microbiota in mother and infant. *Genome Biol. Evol.* 2 53–66. 10.1093/gbe/evp057 20333224 PMC2839348

[B61] Verani J. R. McGee L. Schrag S. J. Division of Bacterial Diseases, National Center for Immunization and Respiratory Diseases, and Centers for Disease Control and Prevention (Cdc). (2010). Prevention of perinatal group B streptococcal disease - revised guidelines from CDC, 2010. *MMWR Recomm. Rep.* 59 1–36. 21088663

[B62] Walther-António M. R. Jeraldo P. Berg Miller M. E. Yeoman C. J. Nelson K. E. Wilson B. A. (2014). Pregnancy’s stronghold on the vaginal microbiome. *PLoS One* 9:e98514. 10.1371/journal.pone.0098514 24896831 PMC4045671

[B63] Yassour M. Vatanen T. Siljander H. Hämäläinen A. M. Härkönen T. Ryhänen S. J. (2016). Natural history of the infant gut microbiome and impact of antibiotic treatment on bacterial strain diversity and stability. *Sci. Transl. Med.* 8:343ra81. 10.1126/scitranslmed.aad0917 27306663 PMC5032909

[B64] Zwittink R. D. Renes I. B. van Lingen R. A. van Zoeren-Grobben D. Konstanti P. Norbruis O. F. (2018). Association between duration of intravenous antibiotic administration and early-life microbiota development in late-preterm infants. *Eur. J. Clin. Microbiol. Infect. Dis.* 37 475–483. 10.1007/s10096-018-3193-y 29368074 PMC5816780

